# Domestic Poultry and SARS Coronavirus, Southern China

**DOI:** 10.3201/eid1005.030827

**Published:** 2004-05

**Authors:** David E. Swayne, David L. Suarez, Erica Spackman, Terrence M. Tumpey, Joan R. Beck, Dean Erdman, Pierre E. Rollin, Thomas G. Ksiazek

**Affiliations:** * U. S. Department of Agriculture, Athens, Georgia, USA; † Centers for Disease Control and Prevention, Atlanta, Georgia, USA

**Keywords:** disease, pathogenesis, poultry, reservoir, SARS-coronavirus

## Abstract

SARS coronavirus injected intratracheally into chickens, turkeys, geese, ducks, and quail, or into the allantoic sac of their embryonating eggs, failed to cause disease or replicate. This finding suggests that domestic poultry were unlikely to have been the reservoir, or associated with dissemination, of SARS coronavirus in the animal markets of southern China.

An outbreak of severe acute respiratory syndrome (SARS) occurred in Guangdong Province, People’s Republic of China, in November 2002 and spread to patients in 30 countries in Africa, Asia, Australia, Europe, and North and South America (*1*,*2*). As of July 11, 2003, SARS had been diagnosed in 8,437 patients; 813 died (*1*). A novel coronavirus was isolated in tissue culture or detected by reverse transcription–polymerase chain reaction (RT-PCR) from multiple respiratory specimens in many patients with SARS (*2*–*4*). The SARS-coronavirus (SARS-CoV) is proposed to be the cause of this syndrome on the basis of its association with human clinical cases (*3*,*4*) and reproduction of pulmonary lesions in experimentally challenged cynomolgus macaque monkeys (Macaca fascicularis) (*5*). Furthermore, some of the first persons identified with SARS-CoV infections were vendors in animal markets of southern China, which suggests a possible animal source (*6*). SARS-CoV has been detected by real-time RT-PCR or isolated from two wild mammalian species, Himalayan palm civet (Paguma larvata) and raccoon dog (Nytereutes procyonoides), in a market in southern China (*7*), but other studies in southern China involving six provinces and Beijing, as well as sampling of 54 wild and 11 domestic animal species, did not find SARS-CoV (*8*). The original source of this virus remains unknown (*3*). The susceptibility of different animal species within the animal meat markets is unknown.

Coronaviruses have been identified in numerous mammalian and avian hosts. Most widely studied and of common occurrence are coronaviruses reported in chickens (Infectious bronchitis virus), turkeys (turkey enteric coronaviruses), cats (feline infectious peritonitis virus and feline enteric coronavirus), dogs (canine enteric coronaviruses), swine (Porcine hemagglutinating encephalomyelitis virus, porcine transmissible gastroenteritis virus, and porcine respiratory coronavirus), cattle (bovine enteric and respiratory coronaviruses), mice (Murine hepatitis virus), rats (sialodacyradenitis virus), rabbits (rabbit coronavirus), and humans (respiratory and enteric coronaviruses) (*9*). However, on the basis of sequence data, SARS-CoV is sufficiently different from these known group 1, 2, and 3 animal and human coronaviruses to be classified as a new group, group 4 coronaviruses (*10*). Most likely SARS-CoV originated from an unknown animal reservoir, not from a benign coronavirus in the human population (*10*,*11*).

Domesticated poultry species are major commodities traded in the animal markets of southern China. Poultry have been shown to be reservoirs for H5N1 and H9N2 avian influenza viruses that have crossed over and caused infections in humans from 1997 to 2003, some with fatal outcomes (*12*–*14*). Therefore, poultry should be examined as potential hosts for infection and amplification of SARS-CoV to determine any potential role they may have played during the emergence of human infections in southern China.

Groups of nine 3-week-old domestic geese (*Anser anser domesticus*), 3-week-old domestic Pekin ducks (*Anas platyrhyncos*), 4-week-old chickens (*Gallus gallus domesticus*), 3-week-old turkeys (*Meleagris gallopavo*), and 5-week-old Japanese quail (*Coturnix coturnix japonicus*) were each injected intratracheally with 10^6.2^ mean tissue culture infective doses (TCID_50_) of Vero E6 propagated Urbani SARS-CoV per bird in a volume of 0.1 mL. The inoculum was the third passage in Vero E6 cells from the original throat swab specimen of the patient. The chickens were specific pathogen–free from an inhouse flock. The other four species were conventional birds obtained at 1 day (geese, turkeys, and ducks) or 5 weeks of age (quail) from commercial hatcheries and raised on site. Oropharyngeal and cloacal swabs were obtained on days 0, 1, 2, 3, 4 and 10 after injection from five birds per group for virus detection by real-time RT-PCR and virus isolation on Vero E6 cells. RNA for RRT-PCR was extracted with the Trizol LS reagent (Invitrogen, Carlsbad, CA) in accordance with the manufacturer’s instructions. Two hydrolysis probe type real-time RT-PCR assays, both targeting the ORF 1b gene, were optimized and run on a Smart Cycler (Cepheid, Sunnyvale, CA) with the superscript platinum taq one-step RT-PCR kit (Invitrogen, Carlsbad, CA). Real-time RT-PCR tests included negative (noninfected tissue culture media, infectious bronchitis coronavirus, and turkey enteric coronaviruses) and positive (Vero E6 propagated SARS-CoV) controls. Two injected birds of each species were euthanized. After necropsy, their tissues were collected for histopathologic examination (all tissue types) and virus detection (plasma, trachea, lung, spleen, kidney, and heart) on days 2 and 4 after injection, and at termination on day 10 after injection. For determination of infection, serum was collected on days 0 and 10 after injection from all birds and tested by indirect enzyme-linked immunosorbent assay for anti-SARS-CoV antibodies. Antigen used to coat plates was tissue culture propagated Urbani strain of SARS-CoV inactivated by γ irradiation (*3*). Secondary “anti-bird” antibody (Bethyl Laboratories, Montgomery, TX) for testing quail and goose serum or plasma, and secondary anti-duck, anti-chicken, and anti-turkey antibodies (Kirkegaard & Perry Laboratories, Inc., Gaithersburg, MD) for testing duck, chicken, and turkey serum and plasma, respectively, were used. Two birds of each species received uninoculated tissue culture fluid and served as the sham-inoculated groups for real-time RT-PCR, standard RT-PCR, virus isolation, and histopathologic and serologic assays.

To determine if SARS-CoV could grow in avian embryos, 9-day-old chicken eggs and 13-day-old turkey embryonating eggs were inoculated by allantoic sac route and 17-day embryonating turkey eggs were inoculated by yolk sac route; all were tested by virus isolation and real-time RT-PCR for SARS-CoV. All laboratory procedures and animal studies were conducted in biosafety level 3 agriculture (BSL-3AG) (*15*) facility with HEPA respiratory protection and barrier clothing procedures for personnel. General care was provided in accordance with the Institutional Animal Care and Use Committee.

To establish the comparative sensitivity of virus isolation and real-time RT-PCR tests, serial dilutions of SARS-CoV propagated in E6 Vero cell culture were tested for virus reisolation in E6 Vero cells and detection of replicase ORF 1b gene by real-time RT-PCR (*16*). Virus isolation was slightly more sensitive, detecting virus in two of three replicates at the 10^-7^ dilution; the real-time RT-PCR test detected SARS-CoV in three of three replicates at 10^-5^ to 10^-6^ dilution, depending on primer sets. The real-time RT-PCR assay detected virus in oropharyngeal swab specimens from two chickens on day 1 PI (Figure). Real-time RT-PCR results were confirmed by standard RT-PCR targeting the same gene (primers: SARS clone1b For 5′- TgACAgAgCCATgCCT-3′, SARS clone1b Rev 5’CAACggCATCATCAgA-3′) and sequencing of the amplified product. No infectious virus was isolated from any of the birds at any time from oropharyngeal or cloacal swab specimens, plasma, or tissues. Histologic examination did not identify any specific lesions. No anti–SARS-CoV–specific antibodies were detected in birds at 0 or 10 days after injection. Levels of SARS-CoV were detected corresponding to the inoculated titers in chicken and turkey embryonating eggs by real-time RT-PCR, but not by virus isolation.

**Figure:**
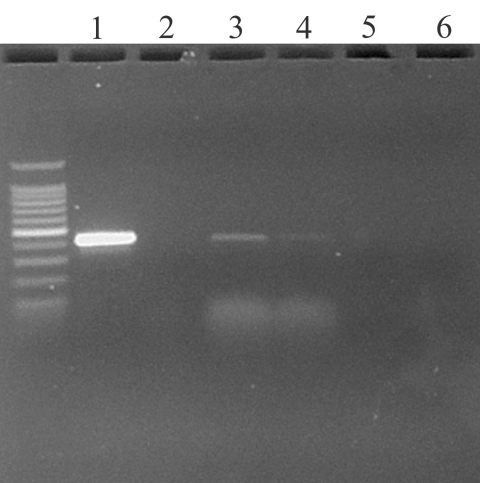
Ethidium bromide stained agarose gel of ORF 1b standard reverse transcription–polymerase chain reaction (RT-PCR) products from oropharyngeal swabs of two chickens day 1 after injection. Key: 1) Positive control (severe acute respiratory syndrome coronavirus from Vero E6 culture); 2) Negative control (water); 3) and 4) Oropharyngeal swabs from chickens 337 and 341 at 1 days after injection; 5) Cloacal swab from turkey at day 2 after injection; and 6) Negative control from cloacal swab of turkey day 0 after injection.

These findings suggest that poultry were unlikely to have been infected during the recent SARS-CoV outbreak and were unlikely to have played any role as amplifiers in the animal markets of southern China. The low level of virus detected by real-time RT-PCR from the chickens and the failure to isolate virus from embryonating chicken and turkey eggs suggest that the detected virus was residual inoculum or nonviable virus and that substantial virus replication in the poultry was unlikely. In addition, this SARS-CoV was of low tissue culture passage, i.e., third passage in Vero E6 cell, which minimized the potential for increased cell culture adaptation and concomitant decrease in vivo replication. Using the original or second tissue culture passage would unlikely have resulted in substantial replication in poultry. However, the virus used in these experiments, the Urbani SARS-CoV, had a 29-nt deletion in the genome. Whether the GZ01 human virus or those from civet cats and raccoon dog containing the extra 29 nt would infect and amplify in poultry would be of interest for future research.
